# Health Star Rating in Grain Foods—Does It Adequately Differentiate Refined and Whole Grain Foods?

**DOI:** 10.3390/nu11020415

**Published:** 2019-02-15

**Authors:** Felicity Curtain, Sara Grafenauer

**Affiliations:** Grains & Legumes Nutrition Council, North Sydney, NSW 2060, Australia; sarag@glnc.org.au

**Keywords:** health star rating, front-of-pack, food labelling, whole grain, refined grain, dietary fibre

## Abstract

The Australian front-of-pack labelling system, Health Star Rating (HSR), does not include whole grain (WG) in its algorithm, but uses dietary fibre (DF), despite Dietary Guidelines recommending WG over refined grain (RG) foods. This study aimed to determine how effectively HSR differentiates WG and RG foods. Product label data were collected 2017–18 from bread, rice, pasta, noodles, flour and breakfast cereals (*n* = 1127). Products not displaying HSR, DF per 100 g, and %WG ingredients were excluded, leaving a sample of 441 products; 68% were WG (≥8 g/manufacturer serving). There was a significant difference (*p* < 0.001) in HSR between WG bread and breakfast cereal over RG varieties, yet the mean difference in stars depicted on the pack was only 0.4 for bread and 0.7 for breakfast cereal. There was no difference for rice (*p* = 0.131) or flour (*p* = 0.376). Median HSR also poorly differentiated WG. More WG foods scored 4–5 stars compared to RG, yet there was notable overlap between 3.5–5 stars. DF content between RG and WG subcategories was significantly different, however wide variation and overlap in DF highlights that this may not be a sufficient proxy measure, raising concerns that the HSR algorithm may not adequately communicate the benefits for consumers of swapping to WG foods.

## 1. Introduction

Nutrition labelling is thought to be an effective policy tool used to promote healthier eating by highlighting a specific quality or nutrient (such as health claims, warnings, or endorsements), or by providing information, such as through a Nutrition Information Panel and ingredients list [1].

In 2014, the Health Star Rating (HSR), a voluntary front-of-pack labelling system, was adopted in Australia and New Zealand, as a joint measure between state and federal governments, public health and consumer organisations, and food industry stakeholders. Like other systems used internationally, the HSR was introduced as a strategy to guide selection of healthier foods in an easily understood manner. The HSR is as an interpretive system that ranks foods on a scale between 0.5 (1/2) to 5 stars, based on the premise that more stars equates to a healthier product [2]. The HSR system was designed to reflect principles outlined in the Australian Dietary Guidelines, and provide consumers with a simple measure to compare foods within the same category [3]. The system uses an algorithm to rate foods on a per 100 g basis, quantifying ‘negative nutrients’, (kilojoules, sodium, saturated fat, and total sugars), and then subtracting ‘positive’ points, based on fruit, vegetable, nuts, or legumes (FVNL) content, as well as dietary fibre and protein content, which is then converted to a star rating. It is worth noting that the FVNL score also includes ingredients such as herbs, spices, fungi, algae and coconut. The HSR algorithm was modified from the existing Nutrient Profiling Scoring Criterion used by Food Standards Australia New Zealand (FSANZ) to determine the eligibility of foods to display health claims, based on their nutritional profile [4]. Currently, the HSR does not directly reward grain foods for containing whole grain through the algorithm, though some whole grain foods may achieve a higher HSR due to the positive points gained from dietary fibre.

Recommendations to include whole grain foods have consistently featured in Australian Dietary Guidelines alongside dietary fibre since their inception in 1979 [5]. This is based on their dietary fibre, protein, B group vitamins, minerals, and antioxidant content compared to refined grain foods, which are altered through processing [6]. Many countries, including the USA, UK, Canada, Denmark, France, Germany, and Singapore also promote whole grain consumption [7,8,9,10,11,12,13]. Observational evidence consistently supports whole grains as a positive dietary component, exhibiting protective effects against all-cause mortality, as well as cardiovascular disease, type 2 diabetes, colorectal cancer, and weight gain [14,15]. Among dietary risk factors globally, low intake of whole grain foods has been noted as the second leading risk for mortality (behind sodium), and the leading risk factor for Disability-Adjusted Life Years (DALYs), leading to 82.5 million DALYs [16].

FSANZ provides a definition for whole grains: ‘the intact grain or the dehulled, ground, milled, cracked or flaked grain where the constituents—endosperm, germ and bran—are present in such proportions that represent the typical ratio of those fractions occurring in the whole cereal, and includes wholemeal’ [17]), but they do not regulate the use of on-pack whole grain claims, as occurs with nutrients such as dietary fibre, protein, energy, vitamins, and minerals [18]. Rather, in 2013, the Grains & Legumes Nutrition Council led the development of the voluntary Code of Practice for Whole Grain Ingredient Content Claims (The Code) in Australia and New Zealand to guide the use of whole grain ingredient content claims on food labels. Cut-off values of ≥8 g whole grain per manufacturer serve (contains whole grain), ≥16 g per serve (high in whole grain), and ≥24 g per serve (very high in whole grain) are stipulated. The Code was designed to complement other recommendations like the Australian Dietary Guidelines and the FSANZ definition for whole grain to aid consumers in meeting the Australian 48 g whole grain Daily Target Intake [19], which was proposed by the Grains & Legumes Nutrition Council in 2006, together with an expert round table, and is consistent with US dietary guidelines [20].

Although some whole grain foods may achieve a higher HSR due to the positive points gained from dietary fibre, the HSR is not unique in excluding whole grain as other front-of-pack labelling systems globally also tend not to reward whole grain content. Reductive systems such ‘warning labels’ seen in Finland, Thailand, Chile, Israel, and Canada merely offer caution on foods with nutrients such as energy, saturated fat, total sugars, and sodium present in substantial amounts, and do not take into account ‘positive’ nutrients or ingredients [21]. The UK Multiple Traffic Lights label, implemented in 2005, displays levels of energy, total and saturated fats, total sugars, and salt, assigning a colour code to the four nutrients—red (high), amber (medium), or green (low), but does not account for positive nutrients or ingredients [22]. The Nutri-Score system, implemented in France in 2017, draws similarities to the HSR, as it relies on a quantitative algorithm to rank products on a qualitative scale, from A, being healthiest, to E, being unhealthiest. Like the HSR, the Nutri-Score considers ‘unfavourable’ content, energy, total sugars, saturated fats, and sodium, and subtracts from these ‘favourable’ content—fruits, vegetables and nuts, dietary fibre, and protein, but also excludes whole grain. Conversely, the Singapore Healthier Choices logo is used to promote healthier foods within categories, and uses nutrient criteria for more than sixty food subcategories, based on both negative additions (total and saturated fats, trans fats, sodium, and total sugar), as well as positive (calcium, dietary fibre, and whole grain) [23]. The Healthier Choices system appears to be unique in considering both dietary fibre and whole grain, with other mentioned front-of-pack systems, including the HSR, only accounting for the former.

More recently concerns have been raised that rewarding foods for dietary fibre, without also recognising that whole grain may not align with Australian Dietary Guidelines that clearly recommend mainly whole grain foods, and may also limit the degree of discernment between refined and whole grain foods. Currently, the suggestion of including whole grain within the algorithm is a key area of consideration in the HSR five-year review, which found whole grain products that are comparably low in dietary fibre scored similarly to refined grain versions, such as brown rice compared with white rice. Similarly, concerns have been raised that awarding points for only dietary fibre does not sufficiently promote natural whole grains over foods with the addition of refined dietary fibres such as inulin [24]. The aim of this study was to determine how effectively HSR differentiates whole grain and refined grain foods.

## 2. Materials and Methods

Product data from bread, rice, pasta, noodles, flour, and breakfast cereals were collected between September 2017–September 2018 from four major supermarkets in the Sydney Metropolitan area (Woolworths, Coles, Aldi, IGA), representing more than 80% of total market share [25], with the addition of a Bakery franchise (Bakers Delight^TM^, Camberwell, Victoria, Australia). Nutrition information was analysed through a recognised process published previously [26].

Data collection for each subcategory involved photographing all on-pack information, including labels, ingredient lists, nutrition and health claims, and Nutrition Information Panels [27]. An additional internet search was conducted to capture any products not available on shelf at the time of data collection, and all data obtained from photographs were then transcribed into a Microsoft^®^ Excel^®^ spreadsheet 2013 (Redmond, WA, USA) for analysis. Data were checked by a second, independent reviewer to detect inconsistencies or errors.

Data for this study were then selected from the larger data set to allow comparison of whole grain and refined grain foods from each subcategory displaying an HSR. Outlined in Appendix A, this included bread (loaves, rolls, sandwich alternatives, and flatbreads), rice (dry and microwaveable, plain and flavoured), pasta (dry, white and wholemeal), noodles (dry and cooked, plain and flavoured), flour (plain and self-raising, white and wholemeal), and breakfast cereals (ready-to-eat, muesli, granola, clusters, hot cereals plain and flavoured).

Products were included in the analysis if the food displayed an HSR on-pack, dietary fibre per 100 g, and percentage of whole grain ingredients, as it is not possible to estimate whole grain accurately without this information. Exclusion criteria are outlined in Appendix B. The remaining grain foods were then categorised into either whole grain or refined grain, based on eligibility for registration with the Grains & Legumes Nutrition Council’s Code of Practice for whole grain claims (>8 g whole grain per manufacturer serve). The mean, standard deviation (*SD*), median and range for dietary fibre (per 100 g), whole grain (per 100 g), and HSR were determined within refined grain and whole grain foods in each subcategory. *T*-tests comparing dietary fibre and whole grain content (g/100 g), and the HSR between whole grain and refined grain subcategories were performed in Microsoft^®^ Excel^®^ 2013 (Redmond, WA, USA).

## 3. Results

**Figure 1:**
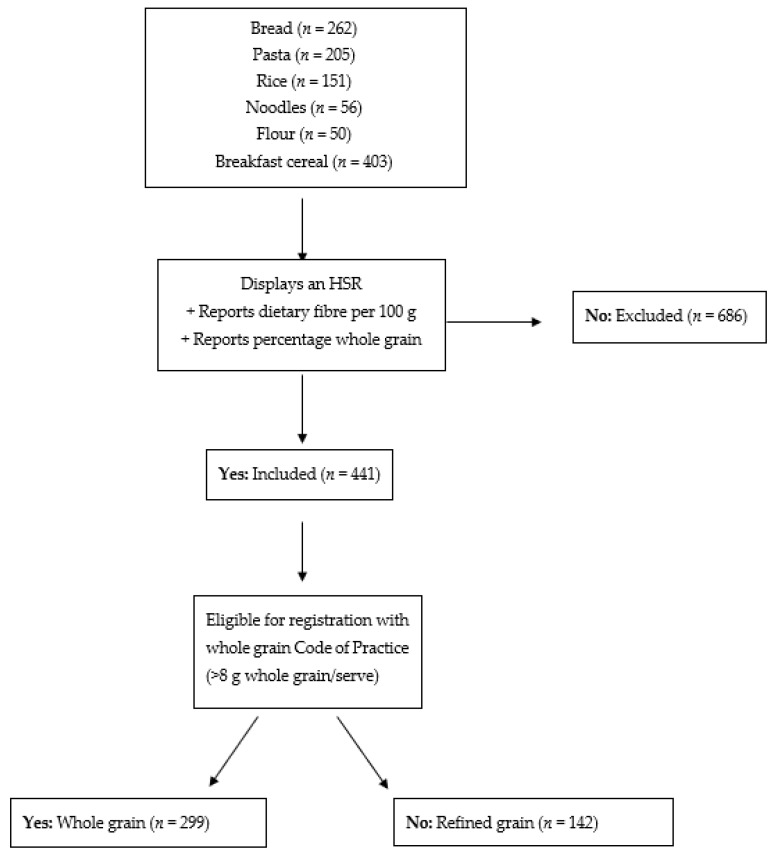
Exclusion criteria for final grain foods sample. HSR: Health Star Rating.

Data from 1127 products were collected, including 262 bread, 205 pasta, 151 rice, 56 noodle, 50 flour, and 403 breakfast cereal products. After excluding products without an HSR, not reporting dietary fibre, and/or percentage whole grain, 441 products remained, of which more than two thirds were whole grain (68%). Figure 1 outlines products meeting selection criteria and breakdown between whole grain and refined grain.

From the total data pool, the breakfast cereal subcategory had the greatest number of grain foods and the greatest proportion of products that met all inclusion criteria (70%). Fifteen percent of pasta, 16% of flour, 17% of bread, and 20% of rice met the inclusion criteria. The exception were noodles, where no products were eligible for inclusion, and were excluded from further analysis. As seen in Appendix B Table A2, the greatest factor in excluding products was the absence of an HSR. While 86% of bread, and 77% of breakfast cereals displayed an HSR, only 27% of rice, 20% of flour, 17% of pasta, and no noodles displayed an HSR.

There were considerably more refined grain products than whole grain within the rice (76%), and flour (75%) categories, while the pasta category had only refined grain products eligible for inclusion. Conversely, there were more whole grain products than refined grain in the breakfast cereal subcategory (83%), and the bread subcategory (54%) (Table 1).

Within whole grain and refined grain subcategories, there was an average 0.4 star difference within breads, 0.3 difference within rice, 0.4 difference within flour, and 0.7 within breakfast cereal, the greatest discrepancy of all subcategories. As seen in Table 2, median HSR values were identical between whole grain and refined grain bread (4.0), a 0.5 star difference was noted between both rice and flour subcategories, and 0.2 difference between flour subcategories. Refined grain breakfast cereal had the greatest range (1.5–5.0) of all refined subcategories, and refined grain bread, rice, and flour each showed a range of 1.5 stars, while refined grain pasta varied between half a star. Whole grain breakfast cereal also had the widest range of whole grain subcategories, between 2.0–5.0 stars. Whole grain bread had the same range as refined grain bread (3.5–5.0), whole grain rice had a smaller range of only 1.0 star, and the two flour products were both rated the same, at 4.0 stars. There was a significant difference (*p* < 0.001) in the HSR for whole grain bread over refined grain bread and for breakfast cereal (*p* < 0.001), but no significant difference for rice (*p* = 0.131) or flour (*p* = 0.376) (Table 2). As HSR is a comparison between foods within the same category, it is not relevant to compare the total whole grain and refined grain categories.

Table 3 outlines the distribution of HSR scores for each subcategory, between 0.5–5 stars. A greater proportion of whole grain foods scored between 4–5 stars than refined grain foods; however, there was substantial overlap in HSR scores between subcategories, as shown in bold in Table 3. For example, almost all whole grain breads scored between 4–4.5 stars (94%), and 89% of refined grain breads scored between 3.5–4 stars. The majority of whole grain rice was rated 4 stars (86%), and 91% of refined grain rice scored between 3.5–4 stars. All whole grain flours scored 4 stars, compared to 50% of refined grain flours, with the remainder scoring 2.5 (16%), and 3.5 (33%). More than half (51%) of whole grain breakfast cereals scored either 4.5 or 5 stars, compared to 23% of refined grain breakfast cereals. However, a similar proportion of whole grain and refined grain breakfast cereals scored 4 stars, 41% and 36%, respectively. Twenty-one percent of refined grain breakfast cereals scored 2 stars, compared to only 1% of whole grain breakfast cereals. Refined grain pasta scored between 4–5 stars; however, no whole grain pasta displayed an HSR, so a comparison was not possible.

Table 4 compares dietary fibre with whole grain within each subcategory and the dietary fibre between the refined and whole grain subcategories. Total whole grain products had on average almost double the dietary fibre found in refined grain foods (9.3 g ± 3.9 g/100 g versus 4.9 g ± 4.3 g/100 g), yet the range of dietary fibre was similar, (0–34.1 g/100 g for whole grain, and 0.2–28 g/100 g for refined grain). Within subcategories, whole grain products were significantly higher in dietary fibre compared with refined grain foods, though the breakfast cereal subcategory, and bread to a lesser extent, appeared unique in that there was a substantially wider range than other subcategories. Whole grain breakfast cereal products ranged between 0–34.1 g/100 g, and refined grain from 1.6–28 g/100 g, while whole grain breads ranged between 2.9–10.8 g/100 g, and refined grain breads between 1.6–15.8 g/100 g. Although whole grain rice (3.0 ± 0.7 g/100 g) was a greater source of dietary fibre compared with refined grain rice (1.4 ± 0.7 g/100 g), it was lower in dietary fibre compared with other subcategories like refined grain bread (4.5 ± 2.9 g/100 g), refined grain pasta (4.1 ± 0.8 g/100 g), and refined grain breakfast cereal (7.3 ± 5.8 g/100 g). From paired *t*-tests, there were significant differences in the whole grain and dietary fibre content within all subcategories.

Foods categorised as whole grain had a greater percentage and range of whole grain (65 ± 25%, and 14–100% whole grain), compared with only small amounts in refined grain products, (0.5 ± 2.4%, and 0–21% whole grain). However, the percentage of whole grain ingredients varied widely in whole grain products, particularly in whole grain bread, (14–70%), and whole grain breakfast cereal, (19–100%), but also to a lesser extent in whole grain rice, (77–100%). The exception to this was whole grain flour, which ranged from 95.5–100% whole grain. Refined grain rice and flour contained no whole grain, and only small amounts of whole grain were seen in refined grain bread (between 0–15%), pasta (0–3%), and breakfast cereal (0–21%).

## 4. Discussion

Front-of-pack labelling systems such as the HSR provide a simple strategy for encouraging healthier food choice, including the promotion of whole grain over refined grain foods. However this study has shown that, by rewarding foods for dietary fibre, and not whole grain, the current HSR algorithm does not adequately differentiate whole grain over refined grain foods.

From the sample of 441 products with an HSR, 68% were considered whole grain. Breakfast cereal made up the largest proportion of products and this was unsurprising as recent survey data found breakfast cereals to be the greatest contributor to whole grain in both Australian adults and children [1].

When comparing whole grain and refined grain subcategories, there was a significant difference in HSR between bread, and breakfast cereal, but not for rice, or flour. However, there was little to no difference in the median HSR in whole grain versus refined grain subcategories. While whole grain products (>8 g/serve) were statistically higher in dietary fibre than refined grain (except for flour), there was a wide range and overlap in dietary fibre noted, which was particularly evident in the breads and breakfast cereals subcategories, and may account for the very minor differences in HSR between refined grain and whole grain choices. Whole grain foods were not consistently higher in dietary fibre compared to refined grain foods, with whole grain rice on average lower in dietary fibre compared with refined grain bread, refined grain pasta, and refined grain breakfast cereal. This was expected, as whole grains are known to vary widely in dietary fibre content, from between 3.5 g/100 g (brown rice), to 18 g/100 g (bulgur) [28,29]. Equally, foods such as bread, breakfast cereals, and pasta may be formulated with added dietary fibre, such as bran, but devoid of the whole grain. The fact that not all high fibre grain foods contain whole grain (and vice versa) highlights a potential issue with the HSR utilising dietary fibre as a surrogate for whole grain, as it is clear that the two measures are not consistently aligned within foods. As a consequence, the current HSR algorithm does not reliably lead to clear differentiation between whole grain and refined grain foods in terms of the stars depicted on pack.

Despite the significant health benefits of naturally occurring whole grains, manufactured foods based on either whole, or refined grains make a meaningful contribution to the diet. In the USA, to address under consumption of nutrients commonly found in whole grains, refined grains such as white flour, white rice, and cornmeal are generally enriched with iron and B vitamins such as thiamin, niacin, and riboflavin, as well as folic acid [30]. Bread flour in Australia has long been fortified with thiamin, to help reduce the incidence of Wernicke–Korsakoff syndrome, and, since 2009, it has been mandatorily fortified with folic acid and iodine (as iodised salt), to address dietary insufficiencies [26]. As a result, grain foods, both refined and whole grain, have been shown to provide a range of shortfall nutrients in the U.S. [31,32]; and, in Australia, data demonstrate that grain foods provide a significant contribution to the intake of B vitamins, iron, calcium, magnesium, selenium, zinc and dietary fibre [33]. 

The Australian Dietary Guidelines and other international dietary guidelines consistently promote whole grain foods for their nutrient density and protection against chronic disease, and yet most Australians fall short of the 48 g Daily Target Intake. Similarly, consumption of whole grain in the UK [34], the USA [35], Singapore [36], France [37], Italy [38], and Malaysia [39] are below recommended intakes. Awareness of Australian Dietary Guidelines is low, with a Western Australian study showing only 6.3% of participants could accurately identify the correct number of servings from the grain foods category. This may be linked with the limited promotion of Australian Dietary Guidelines; with the exception of the government supported ‘Go for 2 & 5’ fruit and vegetable campaign, efforts to increase consumption of core foods have been minimal [40]. Although research conducted in Ireland found awareness of the term ‘whole grain’ was strong, a limited understanding of their health benefits, and limited knowledge of how to identify whole grain foods emerged as major barriers to consumption [41].

Experimental studies have demonstrated the HSR performs well in terms of encouraging consumers to choose products with a higher HSR, and is perceived as simple and easy to use [42,43,44], but there is little consumer research to shed light on how front-of-pack labels are used in the community, or how they impact shopping habits and dietary patterns. In particular, a key area of limited understanding is the subjective nature of which score is considered ‘healthy’, as opposed to ‘unhealthy’, by consumers. In the absence of official guidance, studies have classified ‘unhealthy’ foods below 2 stars, with 2.5 seen as a ‘pass mark’, and above 3 considered healthy. These assumptions are based on findings that the majority of foods rated below 2 were considered ‘discretionary’ as per Australian Dietary Guidelines, and based on consumer perception [44,45,46,47].

Consumer research has also found participants were clearly and rapidly able to judge products with an HSR below 3 as ‘unhealthy’, but mid-range scores were more difficult and time-consuming to interpret [48]. These results are relevant to the grain food subcategories from the current study, as whole grain and refined grain rice, breakfast cereal and flour all included products with scores of 3 and/or below suggesting interpretation may be difficult for consumers. While a greater proportion of whole grain foods from each subcategory scored between 4–5 stars compared to refined grain, there was a notable overlap in scores between 3.5–5 stars, and the majority from both whole grain and refined grain were rated above 3. HSR scores which do not differentiate between refined grain and whole grain varieties may diminish the perceived health benefits of whole grain foods, with only minor star differences unlikely to sufficiently communicate the benefits of swapping to whole grain. This may be a very important factor when consumers are faced with similar products such as in the choice of bread, where most Australians still select white, refined grain varieties over whole grain [49]. Studies investigating how nutrition labels are used have found bread and cereal products to be among the most common foods for shoppers to view labels. This was attributed to the heterogeneous nature of these food categories, highlighting the importance of clear labelling to allow consumers to make better choices at the supermarket shelf [50].

For front-of-pack labelling systems to provide meaning to consumers, widespread uptake is required by the food industry. A recent analysis found uptake of the HSR has been consistently increasing since implementation, with 28% of eligible products in the Australian food supply compliant. Cereal and grain products had the second greatest usage, with 36% displaying an HSR, and 26.7% of bread and bakery products [45]. This was reflected in the current study, which found bread and breakfast cereal were the most likely to display an HSR from the large data pool. Conversely, the current study found no whole grain pasta and no noodle products (refined grain or whole grain) displayed an HSR, limiting the opportunity to communicate the health benefits of products within these subcategories. Furthermore, when considering excluded products, less than half of all rice, flour, and pasta displayed an HSR. Given the voluntary nature, a proposed change to the HSR to include whole grain in the algorithm may provide further incentive for manufacturers of whole grain products to display both the percentage of whole grain ingredients, and an HSR, as products may increase in score on packaging. This incentive could override possible economic drawbacks of adding or altering the HSR.

Strengths of this study include the comprehensive and recent selection of total food product data, collected in the last two years from four major supermarkets in Sydney; however, a number of limitations also exist. The inclusion criteria required foods to display an HSR and report dietary fibre per 100 g, and the percentage of whole grain ingredients, as estimating these is difficult to determine without manufacturer information. The combination of these requirements excluded a large portion of products, and future work focused on obtaining these data to gain a more comprehensive view of the entire grain foods category would be beneficial. Excluding foods without an HSR particularly affected the noodles, pasta, and rice categories. Similarly, products were required to state dietary fibre per 100 g, yet reporting dietary fibre on-pack is not mandatory unless a claim related to dietary fibre is also present on pack. The process of categorising foods as either whole grain or refined grain also relied on the information being included on-pack. As reporting of whole grain in the ingredients list is voluntary, cases of whole grain products with missing data were excluded, reducing the sample size for analysis. Furthermore, the criteria utilised within the Grains & Legumes Nutrition Council’s Code meant that foods with small amounts of whole grain (≤8 g whole grain per manufacturer serve) were categorised as refined grain, therefore this may be more accurately named ‘non-whole grain’. We justified this exclusion criteria, as the HSR system also utilises a cut-off for calculation of the FVNL score, and, considering the current scoring system, it is likely that grain food products containing very small amounts of whole grain would not be recognised. Certain subcategories had small numbers of products (for example, whole grain flour) possibly reducing the generalisability of results for that subcategory.

## 5. Conclusions

Front-of-pack labelling systems such as the HSR pose a simple strategy to encourage healthier food choice, including the promotion of whole grain in preference to refined grain foods. Whole grain and high fibre foods are promoted within dietary guidelines globally as part of a healthy diet, and are a key dietary component linked with protection against chronic disease, yet most populations fall short of the suggested Australian 48 g Daily Target Intake. This study demonstrated the current HSR algorithm results in a significantly higher score between whole grain compared with refined grain foods like bread and breakfast cereal, and that whole grain foods are generally higher in dietary fibre. However, this translated to only minor HSR differences in scores and very similar scores between whole grain and refined grain foods within each of the subcategories examined. A wide variation and overlap in dietary fibre across all grain subcategories, and the finding that not all high dietary fibre foods contain whole grain highlights that dietary fibre may not be a sufficient proxy measure for whole grain. Many high quality whole grain foods were not adequately differentiated over refined grain foods vian HSR. Reflecting whole grain content in the HSR algorithm would help correct this issue and benefit public health by directing consumers to select whole grain foods. Furthermore, acknowledgement of whole grain within HSR may provide an incentive to the industry to innovate and develop more products to fill the whole grain gap in consumption.

## Figures and Tables

**Table 1:** Number and percentage of refined grain and whole grain products within each grain food subcategory.

Subcategory	Refined Grain % (*n*=)	Whole Grain % (*n*=)
Bread (*n* = 63)	46% (*n* = 29)	54% (*n* = 34)
Pasta (*n* = 32)	100% (*n* = 32)	N/A
Rice (*n* = 30)	76% (*n* = 23)	23% (*n* = 7)
Flour (*n* = 8)	75% (*n* = 6)	25% (*n* = 2)
Breakfast cereal (*n* = 308)	17% (*n* = 52)	83% (*n* = 256)
Total: 441	32% (*n* = 142)	68% (*n* = 299)

N/A—nil products in this category.

**Table 2:** Mean HSR for refined and whole grain food subcategories, including standard deviation, median and range.

	Refined Grain				Whole Grain			
	Mean ± *SD*	Median	Range		Mean ± *SD*	Median	Range	*p*-Value
Bread (*n* = 29)	3.8 ± 0.0 *	4.0	3.5–5.0	Bread (*n* = 34)	4.2 ± 0.3 *	4.0	3.5–5.0	<0.001
Pasta (*n* = 32)	4.2 ± 0.3	4.0	4.0–4.5	Pasta (*n* = 0)	N/A	N/A	N/A	N/A
Rice (*n* = 32)	3.6 ± 0.4	3.5	2.5–4.0	Rice (*n* = 7)	3.9 ± 0.4	4.0	3.0–4.0	0.131
Flour (*n* = 6)	3.6 ± 0.6	3.8	2.5–4.0	Flour (*n* = 2)	4.0 ± 0.0	4.0	4.0	0.376
Breakfast cereal (*n* = 52)	3.6 ± 0.5 *	4.0	1.5–5.0	Breakfast cereal (*n* = 256)	4.3 ± 1.0 *	4.5	2.0–5.0	<0.001

Independent samples *t*-test * *p* < 0.001 comparing HSR: Health Star Rating (0.5–5) for refined and whole grain subcategories; *SD*: Standard Deviation.

**Table 3:** Distribution of HSR scores in each subcategory (%).

Grain Food Subcategory	0.5	1.0	1.5	2.0	2.5	3.0	3.5	4.0	4.5	5.0
Bread whole grain (*n* = 34)	0	0	0	0	0	0	3	**62**	**32**	3
Bread refined grain (*n* = 29)	0	0	0	0	0	0	**48**	**41**	7	4
Rice whole grain (*n* = 7)	0	0	0	0	0	14	0	**86**	0	0
Rice refined grain (*n* = 23)	0	0	0	0	4	4.3	**56**	**35**	0	0
Flour whole grain (*n* = 2)	0	0	0	0	0	0	0	100	0	0
Flour refined grain (*n* = 6)	0	0	0	0	16	0	33	50	0	0
Breakfast cereal whole grain (*n* = 256)	0	0	0	1	0	2	5	**41**	26	25
Breakfast cereal refined grain (*n* = 52)	0	0	2	21	0	10	8	**36**	13	10

Pasta refined grain (*n* = 32) excluded as there were no pasta whole grain eligible for inclusion, a comparison was not possible between subcategories.

**Table 4:** Comparison of dietary fibre and whole grain (g/100 g) within whole grain (WG) and refined grain (RG) subcategories and dietary fibre between WG and RG categories.

	Dietary Fibre (g/100 g)			Whole Grain (g/100 g)			
	Mean ± *SD*	Median	Range	Mean ± *SD*	Median	Range	*p*-Value
Bread WG (*n* = 34)	6.1 ± 1.6 *	6.3	2.9–10.8	33.2 ± 18.4	23	14–70	<0.001
Bread RG (*n* = 29)	4.5 ± 2.9 *	3.2	1.6–15.8	1.6 ± 3.4	0.0	0–15	<0.001
Pasta RG (*n* = 32)	4.1 ± 0.8	3.6	3.5–6.4	0.3 ± 0.9	0.0	0–3.0	<0.001
Rice WG (*n* = 7)	3.0 ± 0.7 **	3.2	1.8–4.1	96 ± 7.7	98	77–100	<0.001
Rice RG (*n* = 23)	1.4 ± 0.7 **	1.3	0.3–3.2	0.0	0.0	0.0	<0.001
Flour WG (*n* = 2)	8.9 ± 0.0 **	8.9	8.8–8.9	98 ± 2.2	97.8	95.6–100	0.015
Flour RG (*n* = 6)	3.0 ± 0.7 **	3.3	1.5–3.6	0.0	0.0	0.0	<0.001
Breakfast cereal WG (*n* = 256)	9.9 ± 3.8 *	9.4	0–34.1	67 ± 23	68	19–100	<0.001
Breakfast cereal RG (*n* = 52)	7.3 ± 5.8 *	6.4	1.6–28	0 ± 3.0	0.0	0–21	<0.001
Total WG (*n* = 299)	9.3 ± 3.9	8.9	0–34.1	64 ± 25	66	14–100	<0.001
Total RG (*n* = 142)	4.9 ± 4.3	3.6	0.2–28	0.5 ± 2.4	0.0	0–21	<0.001

Independent samples *t*-test * *p* < 0.05, ** *p* < 0.001 comparing dietary fibre (g/100 g) between RG and WG subcategories.

## Appendix A

**Table A1:** Products included and excluded in the analysis.

Category	Subcategories Included	Subcategories Excluded	Rationale
Bread	Loaves, rolls, sandwich alternatives, flatbreads (*n* = 359)	Bakery breakfast items (e.g., fruit bread, brioche, croissants). (*n* = 97)	Only grain foods, and products with similar nutritional profiles within categories were included
Rice	Dry plain, dry flavoured, microwaveable plain, microwaveable flavoured (*n* = 151)	No products excluded	
Pasta	Dry, white. Dry, wholemeal (*n* = 205)	Gnocchi, fresh pasta, couscous, pulse pasta (*n* = 39)	
Noodles	Dried or cooked, plain or flavoured (*n* = 56)	No products excluded	
Flour	White plain and self-raising, wholemeal plain and self-raising (*n* = 50)	Gluten-free, legume, bread-making, corn, flour blends, non-grain, other grain, rice flour (*n* = 73).	
Breakfast cereals	Ready-to-eat cereals, muesli, granola, clusters, hot cereals plain, hot cereals flavoured (*n* = 441)	Breakfast biscuits, breakfast snacks (*n* = 38)	

## Appendix B

**Table A2:** Meeting inclusion criteria.

Category	% Displaying HSR	Dietary Fibre (g/100 g)	% Whole Grain Able to Be Calculated	All Criteria (HSR, Dietary Fibre, and % Whole Grain)
Bread (*n* = 359)	86%	85%	85%	17%
Rice (*n* = 151)	27%	70%	94%	20%
Pasta (*n* = 205)	17%	70%	97%	15%
Noodles (*n* = 56)	N/A	31%	100%	N/A
Flour (*n* = 50)	20%	60%	98%	16%
Breakfast cereals (*n* = 441)	77%	96%	85%	70%

N/A—no products in this category.
